# Abiotic Stress in Crop Species: Improving Tolerance by Applying Plant Metabolites

**DOI:** 10.3390/plants10020186

**Published:** 2021-01-20

**Authors:** Francisca Godoy, Karina Olivos-Hernández, Claudia Stange, Michael Handford

**Affiliations:** Centro de Biología Molecular Vegetal, Departamento de Biología, Facultad de Ciencias, Universidad de Chile, Las Palmeras 3425, Ñuñoa, Santiago 7800024, Chile; mrgodoy@uc.cl (F.G.); k11.ol.hz@gmail.com (K.O.-H.); cstange@uchile.cl (C.S.)

**Keywords:** drought stress, heavy metal stress, primary metabolite, salt stress, secondary metabolites

## Abstract

Reductions in crop yields brought about by abiotic stress are expected to increase as climate change, and other factors, generate harsher environmental conditions in regions traditionally used for cultivation. Although breeding and genetically modified and edited organisms have generated many varieties with greater abiotic stress tolerance, their practical use depends on lengthy processes, such as biological cycles and legal aspects. On the other hand, a non-genetic approach to improve crop yield in stress conditions involves the exogenous application of natural compounds, including plant metabolites. In this review, we examine the recent literature related to the application of different natural primary (proline, l-tryptophan, glutathione, and citric acid) and secondary (polyols, ascorbic acid, lipoic acid, glycine betaine, α-tocopherol, and melatonin) plant metabolites in improving tolerance to abiotic stress. We focus on drought, saline, heavy metal, and temperature as environmental parameters that are forecast to become more extreme or frequent as the climate continues to alter. The benefits of such applications are often evaluated by measuring their effects on metabolic, biochemical, and morphological parameters in a variety of crop plants, which usually result in improved yields when applied in greenhouse conditions or in the field. As this strategy has proven to be an effective way to raise plant tolerance to abiotic stress, we also discuss the prospect of its widespread implementation in the short term.

## 1. Introduction

**Figure 1:**
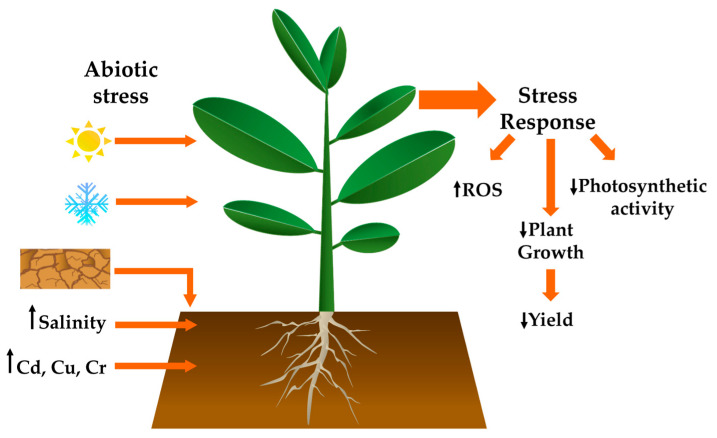
Abiotic stress reduces crop yield. Environmental stress factors, such as heat, cold, drought, salinity, and the presence of heavy metals such cadmium, copper, and chromium, elicit stress responses in plants, including an accumulation of reactive oxygen species (ROS) and reduced photosynthetic activity, which ultimately lower plant growth and thus crop yields.

Abiotic stress is one of the most important problems currently faced by agriculture. It causes serious losses in crop production worldwide and reduces planted acreage. Amidst a growing population and climate change, this scenario becomes increasingly complex. Because the world population is forecast to increase from 7 to 9–10 billion people by 2050, an increase of between 60 and 110% in global food production will be required ([1] and references therein). Arable lands are also affected by migration to cities; as urban areas expand, they encroach more into surrounding, often fertile land, which is another factor that pushes agriculture into areas that are less-suited to crop cultivation [2]. Due to deforestation and the excessive use of fossil fuels, atmospheric CO_2_ levels have increased from 280 to 400 ppm and are expected to rise to 800 ppm by 2100 [3,4]. Furthermore, climate change causes extreme weather, such as brusque temperature fluctuations, extreme precipitation, and drought, among other effects. This is subjecting crop species to increased abiotic stress and impacts production. For example, 45% of arable lands are subjected to drought [5], and the world area under aridity stress increased from 17 to 27% between 1950 and 2000 [6]. Furthermore, salinity accounts for considerable decreases in crop productivity [7], because most crop species are sensitive to salt stress (1.0–1.8 dS m^−1^), and it negatively impacts yield by 10 to 50%, depending on the salt concentration present [8]. Anthropogenic activities, such as mineral extraction and the over-application of fertilisers and pesticides, are also related to increases in heavy metal pollution, including excess hexachromium (Cr), cadmium (Cd), arsenic (As), lead (Pb), copper (Cu), and mercury (Hg). These pollutants affect plant developmental processes, such as seed germination, and the rates of photosynthesis, respiration, and transpiration, thus reducing growth, yield and quality [9,10] (Figure 1). These situations thus demand changes in agricultural practices to respond to the negative impacts of climate change and anthropogenic activities.

Plants are commonly subjected to different kinds of abiotic stress, such as UV, high temperatures, drought, floods, and heavy metals. As sessile organisms, they have evolved structural and metabolic adaptations to survive these conditions, such as increased root area and leaf curling when exposed to drought; greater production of antioxidant compounds such as carotenoids, proline, and ascorbic acid; and higher activity of enzymes related to the scavenging of reactive oxygen species (ROS). Abiotic stress causes an imbalance of pro-oxidant and antioxidant compounds, also known as oxidative stress, via processes that have been covered by excellent reviews [11], including in crop species [12]. Briefly, abiotic stress factors frequently favours stomatal closure, increasing the activity of the photorespiratory pathway, and triggering the production of hydrogen peroxide (H_2_O_2_). Additionally, the diminished uptake and assimilation of CO_2_ results in an imbalance of electron flow through the photosynthetic electron transport chain, again sparking the production of superoxide radicals and singlet oxygen (^1^O_2_). Together with hydroxyl radicals (OH^•^), the excess H_2_O_2_, superoxide radicals and ^1^O_2_ are highly reactive to proteins, lipids, and nucleic acids, damaging them and causing cell death. A common marker for lipid peroxidation, considered one of the most damaging processes, is malondialdehyde (MDA), which is an oxidised product of membrane lipids. To overcome oxidative stress, plants possess enzymatic and non-enzymatic detoxification systems. In the enzymatic system, the enzymes superoxide dismutase (SOD), catalase (CAT), monodehydroascorbate reductase (MDHAR), dehydroascorbate reductase (DHAR), glutathione reductase (GR), glutathione transferase (GST), ascorbate peroxidase (APX), glutathione peroxidase (GPX), and guaiacol peroxidases (POXs) among others, are involved in ROS scavenging. These enzymes present higher activity when plants are subjected to stress. The non-enzymatic mechanism involves the production of compounds such as proline, glutathione (GSH), ascorbic acid, carotenoids, flavonoids, and tocopherols, that alleviate oxidative damage by neutralising ROS [13,14]. In the case of GSH, not only its concentration but the ratio with its oxidised form, glutathione disulphide (GSSG), is important. Crop varieties presenting higher endogenous amounts of some of these compounds display improved tolerance. However, under high intensity or continued stress, these strategies are not enough to alleviate the damage, and plant growth and yield are severely affected.

Currently there are several strategies to increase abiotic stress tolerance in crop species: (1) Genetically engineered organisms (GMOs), including transgenic, cisgenic, and intragenic crops; (2) traditional breeding programs; (3) new breeding technologies (NBTs), including the use of the Clustered Regularly Interspaced Short Palindromic Repeats (CRISPR/Cas) edition strategy; and (4) the application of plant biostimulants. Biostimulants are defined as plant fertilisers that improve nutrient use efficiency, tolerance to abiotic stress, quality traits or availability of confined nutrients in the soil or rhizosphere [15].

Although many GMOs have been bioengineered to tolerate abiotic stress, their practical use depends on the politics and legal aspects of each country, and it is a lengthy process before they are accepted for cultivation and commercialisation. Although more than 500 transgenic crops have been approved for cultivation, only 12 are related to tolerance to abiotic stress due to the high complexity of this trait [16]. Furthermore, there is a generally negative public perception regarding transgenic crops and a lengthy regulatory process to obtain permission for cultivation. It has been estimated that, on average, it costs USD 35 million to process the regulatory safety assessments and authorisations, and that it takes approximately 13 years from the development of a transgenic crop to its commercial launch [16,17]. Regarding genetically edited crops, the CRISPR mushroom was the first edited commercialised food [18], but their governance is still being developed in many countries [19]. Crop varieties obtained by conventional breeding programs are not limited by environmental and biosafety regulations, but present the problem that it takes ten to twenty years to develop a new variety, depending on the species [20].

On the other hand, a non-genetic approach to improve crop yield in stress conditions involves the exogenous application of biostimulants, including naturally occurring plants and fungal and microbial metabolites. For example, the application of amino acids, sugars, secondary metabolites, or artificial compounds has proven to be an effective way to increase plant tolerance to stress, and is relatively easy to implement in the short term. It has been reported that the application of endogenous metabolites enhances the plant stress response, and these compounds have the advantage in that they participate in combating different kinds of stress. Phytohormones, as key regulators of plant development, have also been applied to crops in order to improve tolerance to different kinds of abiotic stress. Exogenous application of auxin, cytokinins, abscisic acid, salicylic acid, and gibberellin have all proven useful in alleviating the effects of abiotic stress, as have transgenic means to enhance endogenous hormone production ([21,22,23,24,25] and references therein). As the role of phytohormones in abiotic stress has been widely described by other colleagues in the aforementioned publications, this review will focus on primary and secondary metabolites.

It is known that in plants, fungi, and microorganisms, more than 200,000 substances are produced, and they can be grouped into primary and secondary metabolites [26]. Primary metabolites are synthesised by all organisms and are essential for central, vital processes such as growth and development, and include proteins, lipids, and carbohydrates. In plants, secondary metabolites are synthesised in different cellular compartments, and include phenolics, terpenoids, and alkaloids as main groups, whose many functions include protection against abiotic and biotic stress. Different metabolic routes are involved in the synthesis of these molecules, such as the shikimate pathway, the acetate–malonate pathway, and side reactions emanating from glycolysis and the tricarboxylic acid (TCA) cycle [26,27,28]. Compared with primary metabolites, secondary metabolites are produced in low levels (<1% dry weight) and are specific to certain organs or developmental stages.

In this review, we present the latest literature on how the exogenous application of key natural primary and secondary metabolites has been used to improve abiotic stress tolerance (salinity, drought, and heavy metal) in both greenhouse and field conditions, with a focus on their effects on growth, physiological parameters, and yield in crop species. Exogenous metabolite application is an easy-and-feasible-to-implement strategy for producers, although the time interval in which they exert their beneficial effects depends on dose, the time of application and crop species. Finally, we comment on the challenges to overcome so that growers and producers can profit from the knowledge generated in this fast-developing area.

## 2. Primary Metabolites

### 2.1. Proline

Proline is one of the most studied osmoprotectants in plants. In 1954, Kemble and McPherson described proline accumulation in wilting rye grass (*Lolium perenne*), and since then, it has been the focus of many studies. Proline can function as a molecular chaperone, protecting protein integrity. Moreover, it acts as a singlet oxygen quencher, contributing to the maintenance of redox balance [29,30]. However, the beneficial effects observed in abiotic stress tolerance depend on species, concentration, phenological time of application, and application system.

Extensive literature exists on the beneficial effects of proline when applied to crop species under abiotic stress. For example, Teh et al. (2016) [31] applied proline (5 and 10 mM) to 30-day-old rice (*Oryza sativa*) plants cultivated in vitro under saline stress; the higher concentration significantly increased plant height and shoot and root number when compared to control stressed plants. Additionally, in rice, Tabssum et al. (2019) [32] observed improved water relations in salt+proline-treated plants grown in greenhouse conditions, as well as an increase in SOD and CAT activities, indicating an enhanced effect of proline on ROS scavenging when compared to salt-stress conditions. However, 50 mM-proline treatments had adverse effects on bean (*Vicia faba*), where plant growth and photosynthetic pigment accumulation were similar to that of the sea water treatments [33], indicating toxic effects. Thus, dose optimisation is a crucial step towards its use for yield improvement in agriculture.

It has been reported that proline treatment in the context of abiotic stress induces changes at the structural and ultrastructural levels. An increase in root surface is one of the strategies adopted by plants to overcome nutrient or water shortage. Proline application during salt stress induced changes in root surface in rice [31], increasing the number of roots per plant. Regarding the aerial part, proline application in salt-stressed plants produced structural changes in stems and leaves. Rady et al. reported an increase in cortical layers, xylem vessel diameter, and cortex thickness in two varieties of lupine (*Lupinus termis*) grown in salinity conditions, when 3, 6 or 9 mM proline was foliar-applied at 20, 35, and 50 days after sowing, and the best results were obtained with a proline concentration of 6 mM [34]. At the ultrastructural level, treatments with proline in rice leaves under control conditions produced swelling of thylakoids and an increase in total starch area. Salt stress also had this effect, especially 100 mM NaCl. However, when proline was applied in rice plants under saline stress, chloroplasts maintained their structural integrity, and no thylakoid swelling was observed. This is consistent with reports describing a protecting role of proline on thylakoid membranes by scavenging ROS [35].

The effectiveness of proline in the alleviation of metal toxicity has also been studied. Treatment by excess caused a decrease in growth parameters, an increase in MDA and H_2_O_2_ levels, and SOD and POX activities in eggplant (*Solanum melongena*). However, As+proline treatment caused an increment in dry weight (DW) and root length, reduced As content in eggplant seedlings, decreased H_2_O_2_ and MDA levels, and increased activity of ROS scavenging-related enzymes. It also generated higher activity levels of the two proline biosynthesis enzymes, Δ1-pyrroline-5-carboxylate synthetase (P5CS) and proline dehydrogenase [36]. Likewise, in rice treated with Cr, proline application significantly decreased MDA levels [37]. In a pot experiment where wheat (*Triticum aestivum*) was subjected to Cu stress, proline application increased yield (100-grain weight), although it depended on the cultivar used [38].

Further studies evaluating crop yield improvement under field conditions and long-term stress are needed in order to validate the use of proline by producers.

### 2.2. L-Tryptophan (TRP)

Auxin is the most studied hormone in plants. Because abiotic stress impairs plant development and growth, one of the approaches to overcome this has been the application of L-tryptophan (β-3-indolylalanine), an essential amino acid that serves as precursor for auxin biosynthesis [39].

The effect of TRP in ameliorating salt-induced damage in crops has also been studied. Red peppers (*Capsicum annuum*) grown in pots in a wire house were subjected to different salinity levels and TRP treatments. Plant growth parameters, such as plant height, root length, shoot, and root DW, increased in all conditions when TRP was applied, as did chlorophyll and carotenoid levels. Moreover, fruit weight was higher when compared to untreated controls. At high salinity, control plants did not produce fruit; however, under TRP treatment, plants were able to develop fruits, albeit small ones [40]. Other studies carried out in sugar beet (*Beta vulgaris*) showed that several root parameters at harvest were improved in plants subjected to different salinity levels whose seeds had been pre-soaked with TRP when compared to salt-stressed plants which had not been treated with this amino acid [41]. Furthermore, salinity decreased root quality by increasing K^+^, Na^+^, and α-amino-N, which are considered impurity parameters. TRP seed treatment improved said parameters, thus enhancing root quality [41].

Regarding drought stress, TRP has proven to improve yield. Jamil, 2018 [42], performed a pot experiment, where wheat (*Triticum aestivum*) was subjected to different irrigation treatments. TRP was foliar sprayed every week, starting 15 days after germination and ending two weeks after the filling stage. TRP treatment increased growth parameters, such as plant height, root length, and root DW, in all irrigation conditions when compared to control plants, as well as the amount of photosynthetic pigments. TRP also increased yield, either under control or drought conditions [42].

Moreover, TRP improves yield in crops subjected to heavy metal stress. In rice subjected to Cd stress, a decrease in plant growth (plant height and number of tillers) and yield parameters (number of panicles, 1000 grain weight, and paddy yield) was observed when compared to control plants. However, Cd+TRP treatment increased those parameters when compared to plants subjected to Cd stress alone [43], indicating that TRP is effective at alleviating Cd-induced damage.

Hanci et al. evaluated the effect of TRP on seed germination under temperature stress [44]. They treated onion (*Allium cepa*) and leek (*Allium porrum*) seeds with different concentrations of TRP, and subjected them to 7, 21, and 35 °C in a germination chamber for 21 days. In onion, germination percentage at the lowest temperature was slightly increased by TRP treatment (125 ppm), although higher doses of this metabolite decreased the germination rate. In leek, beneficial effects of TRP were observed only in cold stress conditions (7 °C). The authors conclude that although TRP application may improve germination, further studies are needed to identify the appropriate dose for each species [44].

In summary, TRP has been seen to induce plant growth and yield parameters in crops subjected to different kinds of stress, such as salt, drought, and excess Cd, although the cost of applying in the field must be considered and evaluated.

### 2.3. Glutathione (GSH)

GSH (γ-l-glutamyl-l-cysteinylglycine) is a ubiquitous low molecular weight tripeptide, composed of the essential amino acids glutamine, cysteine, and glycine [45], although some plants exhibit a variation called homoglutathione, with the same biological properties. It is present in most plant tissues, and among its major functions are ROS detoxification, formation of phytochelatins that bind heavy metals, detoxification of methylglyoxal, and its role as a cysteine reservoir [46,47].

GSH application in mungbean (*Vigna radiata*) seedlings grown under salt stress in growth chambers improved the relative water content (RWC) and chlorophyll (*a* and *b*) levels at 24 and 48 h after GSH treatment. Classic stress marker levels such as H_2_O_2_, MDA, and superoxide generation rate were also significantly decreased in the salt+GSH treatment when compared to the salt stress treatment alone. The GSH/GSSG ratio was also significantly increased when comparing GSH treatment to salt stress alone, indicating a better redox state. Enzyme activities from the ROS scavenging system (SOD, APX, GST, MDHAR, DHAR, GR, and GPX) were also increased in plants subjected to salt+GSH when compared to control plants under salt stress alone [48]. Zhou et al. also evaluated the enzyme activity and transcript abundance of ROS scavengers under GSH and salt treatments in tomatoes (*Solanum lycopersicum*) grown under greenhouse conditions [49]. They found that salt stress decreased the activity levels of SOD, POD, CAT, MDHAR, DHAR, and APX, as well as GSH, when compared to control conditions, but GSH treatment improved enzyme activity when compared to salt stress. The foliar application of GSH to tomato seedlings grown under salt stress reduced H_2_O_2_, MDA, and superoxide generation rate levels, as well as a decrease in Na^+^ and Cl^-^ uptake and accumulation when compared to salt-stressed plants [49]. Even though the study evaluated the effect of GSH at 5, 10, and 15 days after treatments, the results were consistent with those reported for other species [48,49].

Moreover, eleven soybean (*Glycine max*) varieties grown under nets showed significant increments in plant growth and production parameters when GSH was applied to leaves of salt-stressed plants in comparison to stress-only conditions [50]. For example, the number of seeds per plant increased by 22–62% (depending on the genotype examined) when compared to stressed treatment. Furthermore, other parameters interesting for their impact on crop yield were significantly improved, such as pods per plant (by 12–60%) and yield per plant (by 16–67%), when compared to plants subjected to salt stress alone [50]. Finally, genotypes categorised as susceptible to salt stress presented better responses when also treated with GSH [50], emphasising the importance of conducting studies of the effects of exogenous metabolite application for each genotype. Such studies will be very important for encouraging the cultivation of salt-susceptible varieties with desirable or unique commercial traits, even in saline soil conditions.

Given the importance of GSH as a precursor for phytochelatin synthesis, its effect on alleviating heavy metal toxicity in crops has been evaluated. Khan et al. reported that Pb toxicity decreased photosynthetic attributes such as photosynthetic rate, stomatal conductance, intercellular CO_2_ concentration, transpiration rate, and photosynthetic pigments, and also increased MDA, H_2_O_2_, and Pb levels in upland cotton (*Gossypium hirsutum*) grown in hydroponic conditions [51]. GSH applied to the nutrient medium improved the aforementioned parameters when compared to Pb-treated plants, reaching in many cases the levels present in control conditions. It also amended Pb-induced damage observed at the ultrastructural level, maintaining the integrity of chloroplasts and other organelles [51]. Additionally, in a study conducted by Kim et al. [52], GSH improved seed germination and seedling growth in tobacco (*Nicotiana tabacum*), red pepper and even a model Brassicaceae species (*Arabidopsis thaliana*) under Hg stress. They also demonstrated that GSH presented stronger binding affinity to Hg when compared to other heavy metals such as Cd, Cu, or zinc [52], indicating that GSH may be better suited to alleviate Hg-induced damage.

GSH application also had positive effects on plants subjected to high temperatures. Mungbean seedlings grown in growth chambers were exposed to 42 °C or normal conditions (25 °C) and pretreated with GSH 24 h before the high temperature treatment. Leaf RWC, chlorophyll, and proline content increased in plants treated with GSH when compared to the plants subjected to high temperature. The stress markers MDA and H_2_O_2_ content, as well as superoxide generation rate, were diminished in GSH-treated plants when compared to the stress treatment. Additionally, the ascorbic acid content and GSH/GSSG ratio were increased in GSH-treated plants in comparison to the levels found in heat-stressed plants [53].

### 2.4. Citric Acid (CA)

Citric acid (CA), the initial intermediate of the TCA cycle, is synthesised by citrate synthase from the condensation of oxaloacetate and acetyl CoA [54]. This molecule is a mild antioxidant that can also quelate metals such as Cu, Pb, and aluminium [55]. Problems induced by salinity can be alleviated by CA application. For instance, the addition of CA to the irrigation solution of tomatoes in calcareous soils (high concentration of CaCO_3_) improved the uptake and assimilation of Zn, Na, Ca, and N in leaves, and Mn, Na, Mg, and P in fruits [56]. Likewise, maize (*Zea mays*) plants grown in soil with high salinity and 100–200 ppm CA showed restored height, chlorophyll *a* and *b* contents, enhanced growth, and an improvement in yield when compared to salt-treated controls [57].

Regarding heavy metal stress, exogenous applications of CA to mustard (*Brassica juncea*) leaves reduced the harmful effects of Cd by improving the enzymatic antioxidant response, via increased CAT, POX, and SOD activities. Additionally, the chlorophyll content and stomatal conductance and aperture all rose, leading to a 14% increase in CO_2_ availability, improved photosynthetic rate (by 42%) and growth (by 12%) of the Cd+CA plants compared to mustard stressed with Cd [58].

On other hand, the application of CA can revert the reduction in germination, mineral absorption, hormone homeostasis, and lower growth and yield produced by Pb stress. In the case of castor bean (*Ricinus communis*) grown in soil with 600 mg kg^−1^ Pb and with applications of 5 mM CA, an improvement was observed in the length of the roots and shoots and the number and area of leaves per plant compared to the plants grown in soil with Pb alone, resulting in phenotypes similar to those of control plants (without application of Pb or CA). The photosynthetic parameters, transpiration rate, water use efficiency, and stomatal conductance were improved, as were chlorophyll *a* and *b* and carotenoid levels. The activities of antioxidant enzymes, such as POX, CAT, APX, and SOD, were also enhanced in both roots and leaves of Pb+CA plants [59].

Another case is the stress inflicted by Cu, a metal cofactor for many enzymes involved in electron transfer and an essential micronutrient. Nevertheless, at higher concentrations, it has a toxic effect on seed germination, photosynthetic activity, and cell division; induces changes in the ionic form of nutrients; and generates oxidative stress. It has been shown that canola (*Brassica napus*) can tolerate soils contaminated with an excess of Cu, but plant growth, shoot and root length, and photosynthetic pigments all suffer decreases, whilst the activity of antioxidant enzymes increases, affecting yield. However, the application of CA can counteract this effect [60]. Specifically, the application of 2.5 mM CA in soil together with 100 µM Cu recovered growth and increases of 20–31% were observed in shoot and root length and the number and area of leaves when compared with Cu (100 µM) alone. Similarly, the photosynthetic pigment content and the activities of antioxidant enzymes (SOD, CAT, POX) were raised in Cu+CA-treated canola compared to the Cu-treated controls. A similar finding was discovered in pea (*Pisum sativum*), in that the application of 100 µM CA in the Cu-containing germination medium restored biomass production in roots and shoots, reduced cell death, and corrected cellular redox state versus seeds treated with Cu alone [61]. In white jute (*Corchorus capsularis*), a plant that is used in phytoremediation due to its tolerance to heavy metals, the application of 2 mM CA in the solution media improved parameters like plant growth, biomass, chlorophyll, and carotenoid contents, whilst lowering the activities of SOD and POX [62].

Cr, another heavy metal, can become toxic to plants in high concentrations. In plants, the stress induced by Cr diminishes seed germination, growth, uptake of mineral nutrients available in the soil, and photosynthesis. Such effects can be reverted by the exogenous application of CA. In rice plants, supplementing the irrigation solution with 100 µM CA restored parameters like root and shoot length and DW. Rice plants also had higher internal Cr concentrations (by 50%), demonstrating the capacity of CA to chelate metals, mirrored by enhanced activities of the detoxification enzymes (CAT, POD, SOD, and GR) and metabolites (GSH and proline), enabling the Cr+CA plants to cope better with scavenging ROS [63]. Similar findings have also been discovered in Cr+CA-treated spinach (*Spinacia oleracea* [64]).

**Figure 2:**
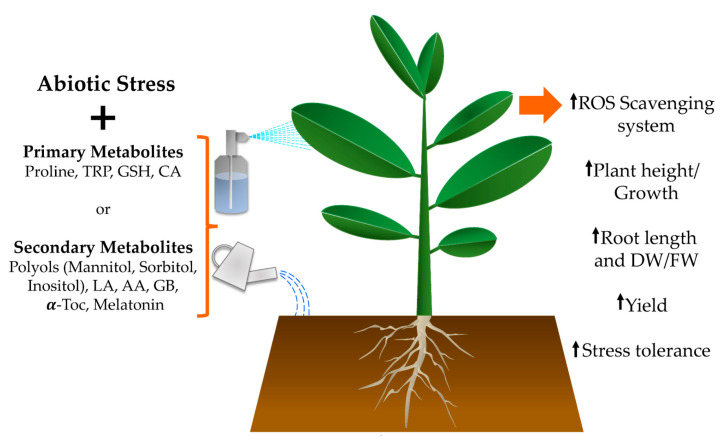
Application of primary and secondary plant metabolites ameliorates the negative effects of abiotic stress. By applying primary metabolites such as proline, tryptophan (TRP), glutathione (GSH), and citric acid (CA), or secondary metabolites like polyols, lipoic acid (LA), ascorbic acid (AA), glycine betaine (GB), α-tocopherol (α-Toc), and melatonin as foliar sprays or in irrigation water, the tolerance of crops is enhanced when faced with environmental challenges.

The multiple effects of adding the aforementioned primary metabolites are summarised in Figure 2, and the principles of their modes of action also hold true for studies that have evaluated the consequences of applying secondary metabolites to different crop plants in greenhouse and field conditions.

## 3. Secondary Metabolites

### 3.1. Polyols

Polyols or sugar alcohols are organic compounds derived from sugars, in which the carbonyl group (CO) has been reduced to an alcohol (OH). They include linear and cyclic forms, termed alditols and inositols, respectively. Examples of polyols are mannitol (reduced form of mannose), sorbitol (from glucose), and inositol, synthesised from glucose 6-phosphate followed by dephosphorylation [65,66,67].

#### 3.1.1. Mannitol

Protective effects on maize with foliar applications of mannitol (15 and 30 mM) have been observed under saline stress (100 mM NaCl). The combined treatment increased total plant DW, RWC, and chlorophyll content (*a* and *b*), demonstrating that mannitol was mitigating, directly and/or indirectly, the harmful effects of saline stress and enhanced biomass production due to its role in cellular osmotic adjustment [68]. Such beneficial properties have also been discerned in the field: the reduced productivity seen in saline-stressed cabbage (*Brassica oleracea*) was alleviated by the foliar application of 2–4 g L^−1^ mannitol [69].

As mentioned above, Cr stress affects several important parameters of crops. The application of mannitol to pot-grown maize can alleviate these effects. The height, leaf area, root length, number of leaves per plant, and DW of all plant parts were improved by the foliar application of 50 and 100 mg L^−1^ mannitol to cultivars under Cr stress (5 and 10 mg kg^−1^ soil) when compared to plants without mannitol, and phenotypes were similar to control plants (without Cr or mannitol). Oxidative stress levels in roots and leaves were also lowered, especially in a more-tolerant cultivar, as well as the activity of antioxidant enzymes (SOD, POD, CAT, and APX) and the content of chlorophylls and carotenoids, suggesting a protective role of mannitol in maize under heavy metal stress [70]. Even though the application of 100 mM mannitol alone might have negative effects on wheat growth under normal conditions, because of induction of stomatal closure, when this polyol was combined with Cr (0.25 and 0.5 mM), pot-grown plants had enhanced shoot and root DWs, as well as husk and kernel weights, facilitated by increases in antioxidant systems in both leaves and roots [71].

Boron (B) toxicity is another kind of stress that has been observed mainly in arid and semi-arid environments, such as those found in South Australia, Turkey, California, and Chile. At high concentrations, B causes necrotic lesions in leaf tips and patches on leaves, reducing photosynthesis, growth and yield [72]. Two wheat cultivars, with different sensitivities to B, were analysed under increasing B concentrations (0, 30, 45, and 60 mg kg^−1^) and mannitol treatments (0, 1, 5, and 10 g kg^−1^) in pots. It was observed that the content of B in leaves was higher in the sensitive cultivar, and that application of mannitol reduced considerably the B content in both cultivars. In this crop, it was determined that 1 g kg^−1^ mannitol was more effective than the other concentrations in reducing the symptoms of B toxicity [73].

#### 3.1.2. Sorbitol

The eggplant is a crop classified as moderately sensitive to salinity. To mitigate the damage caused by salt, the effects of foliar applications of sorbitol (5 and 10 mg L^−1^) to plants in greenhouse conditions subjected to different NaCl treatments (1.5, 3, and 6 dS m^−1^) was studied. It was observed that 5 mg L^−1^ sorbitol was the most efficient, increasing parameters like plant height, the number, weight, and diameter of fruits, as well as chlorophyll *a* and *b* and carotenoid levels in all salt treatments, compared to the controls (without sorbitol) [74]. Studies in rice [75] and wheat [76] have uncovered similar effects, although finding the correct concentration is key for success. For example, in rice, 10 mM sorbitol lowered H_2_O_2_ and MDA levels in 170 mM NaCl-treated plants, increasing growth and DW [75], whereas in wheat, either 50 or 100 mM sorbitol had particularly beneficial effects on root and shoot lengths and DW, proline content, and photosynthetic pigment levels [76].

#### 3.1.3. Inositol

The application of this cyclic polyol has been used to improve abiotic stress tolerance in a few cases. For instance, pre-treating crab apples (*Malus hupehensis*) with 50 µM myo-inositol in the hydroponic media reduced salt stress (200 mM) symptoms, as determined by improvements in plant height and DW, as well as root length, volume and surface area, compared to plants subjected to salt stress, whilst lowering relative electron leakage, H_2_O_2_ production, and Na+ accumulation [77].

In the case of drought stress, exogenous myo-inositol can reduce its harmful effects by protecting the water status of leaves. It was observed that foliar application of 5, 15, and 25 µM myo-inositol to pepper plants under drought stress (7 days without watering) in pots raised the RWC and reduced H_2_O_2_, proline and MDA levels [78].

### 3.2. Lipoic Acid (LA)

Lipoic acid (LA, 6,8-thioctic acid or 1,2-dithiolane-3-pentanoic acid) is a powerful sulphur-containing antioxidant, whilst also acting as a cofactor in several multienzyme complexes involved in primary metabolism [79].

The antioxidant properties of LA have been used to benefit crop production. For example, in canola under salinity stress (<150 mM NaCl), the foliar application of 100 µM LA reduced lipid peroxidation levels, and raised cysteine content and POD and CAT activities, improving overall yield and providing tolerance to salt stress [80]. Additionally, in pot-grown wheat irrigated with different dilutions of seawater (<14.6 dS m^−1^), the foliar application of 0.1 mM LA buffered the deleterious effects by increasing levels of proline, the RWC, and activities of antioxidant enzymes (such as CAT, SOD, POX, and APX), which benefited agronomic parameters (leaf area, plant height, and grain yield) and improved tolerance to salt stress [81].

In the case of drought stress, exogenous application of LA also can be beneficial to plants, as shown in hydroponically propagated maize. Supplementation of the nutrient solution with PEG6000 simulates water deficit. However, the addition of 12 µM LA in the growth media can reduce lipid peroxidation and increase RWC, endogenous LA, chlorophyll *a* and *b* levels, and the expression of key genes (*rubisco large* and *small subunits* (*LSU* and *SSU*), whilst also improving stomatal conductance and photosynthetic and transpiration rates compared to plants treated with PEG6000 alone [82].

Similar effects occur in Pb-treated (1.5 mM) wheat seeds, imbibed with 2 µM LA; these had longer roots and coleoptiles, even though both organs contained more Pb than seeds not treated with LA. The altered ratio of GSH/GSSG demonstrated that the LA-treated plants had a greater capability to withstand the potentially toxic effects of internal Pb [83].

These examples of the use of LA clearly highlight how the combination of species and abiotic stress influences the dose of the metabolite required to alleviate negative effects (100 µM canola/saline; 12 µM maize/drought; 2 µM wheat/Pb).

### 3.3. Ascorbic Acid (AA)

Ascorbic acid (AA), or vitamin C, is another secondary metabolite which can be synthesised de novo from several pathways, such as D-glucose, L-galactose, uronic acid, L-gulose, and myo-inositol pathways, or by recycling its oxidised form. It is a cofactor for many enzymes, and can neutralise ROS, repair oxidised organic molecules, and regulate physiological processes (such as cell division, growth, development, and stress tolerance) in plants [84,85,86].

The application of this metabolite can improve the tolerance of many species to a variety of abiotic stresses. In the cereal crop, quinoa (*Chenopodium quinoa*), different intensities of drought stress reduce quinoa shoot and root length, DW, levels of chlorophylls *a* and *b*, and total carotenoids, whilst raising H_2_O_2_ and soluble sugar levels. However, when 150 mg L^−1^ AA was applied to leaves during the vegetative stage in greenhouse conditions, tolerance to drought stress was dramatically enhanced, as demonstrated by improvements in metabolic, biochemical, morphological and production parameters [87]. Similarly, for two pot-grown varieties of peach tree (*Prunus persica*), growth, nutrient uptake, yield, and fruit quality were all favoured by spraying leaves with 250 ppm AA subjected to water stress [88], as a consequence of higher net CO_2_ assimilation rate, stomatal conductance, proline levels, and antioxidant enzyme activities. Such positive effects have also been seen in the field: the application of AA to wheat leaves (200 mg L^−1^) over two seasons in an experimental farm increased the relative water and chlorophyll content, with higher antioxidant enzyme activities (CAT and POX), resulting in improvements in the number of grains per spike, the number of spikes, and, consequently, yield [89].

In wheat under stress by Pb (2 mM), the application of 0.6 mM AA in the watering solution restored parameters such as root and shoot length; fresh weight (FW); the content of N, P, K, Ca, Mg, and cysteine; and total chlorophyll content, allowing the plant to develop with no serious consequences on yield, thus resembling treatment controls (without Pb or AA) and performing better than Pb-treatment alone. The antioxidant effect of AA also protected the chloroplasts from oxidative damage, as well as altering the concentration of plant hormones such as auxin, gibberellins, and abscisic acid [90].

Heat is another stress that affects the defence system of plants. For example, cotton is cultivated in various climates, but is sensitive to extreme temperatures, although even minor variations in the temperature can reduce the photosynthetic rate and generate ROS. However, the foliar spray application of 70 ppm AA to cotton cv. AA-802 can enhance the activity of SOD and CAT, raise chlorophyll content and antioxidants, and alleviate oxidative stress to such an extent that fibre lengths and, thus, the yields of the plants are affected to a much lesser extent or may even improve compared to the controls [91]. Such results are similar to those discovered in a previous study [92] in which the application of different concentrations of AA to cotton plants during the squaring and flowering stages under heat stress enhanced their RWC, total chlorophyll, the activity of antioxidant enzymes (POX, CAT, and SOD), and the yield compared to controls sprayed with water.

At the other extreme, low temperatures (chilling stress) can also affect crops, causing phenotypic changes such as chlorosis, wilting, metabolic changes, and the leakage of cellular solutes. An example of this can be seen in tomato plants stressed by chilling (4 °C), where leaf area, length, and width are reduced [93]. However, chilled seedlings incubated in AA solution (0.5 mM for 48 h) behaved similarly to the non-chilled controls, showing reduced oxidative damage and increased proline, chlorophyll, mineral nutrition, and expression of molecular chaperones (HSP70, HSP90, and HSP80) and CAT genes [93]. Likewise, AA application can also aid fruits, even after harvesting. In banana (*Musa* spp), chilling affects many phenotypical characteristics important for consumers, such as the colour of fruit peel and pulp, that hinder the sale of the product. Yet a simple procedure of immersing the fruits in a solution of AA at different concentrations (the best was 9 mM) followed by cold storage (27 days at 6 °C) reduced the overall chilling injury index score, reflected in improvements in total chlorophyll, total phenolic and flavonoid content, and the activity of antioxidant enzymes (APX, POD, SOD, and CAT) [94]. AA applications can even improve extreme chilling (freezing) tolerance, as seen in assays with spinach [95].

### 3.4. Glycine Betaine (GB)

GB (N,N′,N′′-trimethylglycine) is an amphoteric quaternary ammonium compatible osmolyte that confers tolerance under environmental stress [96,97]. Its main beneficial effects are through osmotic adjustment and stabilisation of the photosystem II complex, amongst others [96,98]. Not all plants are able to accumulate GB under abiotic stress, although crops and cultivars that produce higher amounts of GB are usually more tolerant to abiotic stress [97].

Foliar application of GB to safflower (*Carthamus tinctorius*) plants subjected to salinity stress and grown under greenhouse conditions induced higher chlorophyll *b* and carotenoid levels, as well as a higher Fv/Fm rate. Moreover, a decrease in Na^+^ levels in leaf tissue was observed, indicating that GB application has positive effects in the response of safflower to abiotic stress [99]. Exogenous application of GB in lettuce (*Lactuca sativa*) grown under salt stress and greenhouse conditions also resulted in higher tolerance to salinity, reflected in increased DW, organic acid, and amino acid levels when compared to control plants [100].

Application of GB to enhance abiotic stress tolerance on plants grown under field conditions has also been promising. In pea, GB application ameliorated the effects of drought stress by increasing yield and soluble protein concentration. This study evaluated short- and long-term drought stress, at different developmental stages, generating comparable scenarios to those found in field conditions [101]. In wheat, a field study using 19 genotypes grown under prolonged drought stress showed that foliar applications of this metabolite improved flag leaf net photosynthetic rate and stomatal conductance when compared to stress conditions alone. Although significant differences in chlorophylls and carotenoid accumulation were not observed, increases in AA were detected when compared to plants subjected to drought. However, responses to GB were genotype specific [102]. However, the effects of GB may be species specific—for example, no improvements were observed in growth or yield when applied to tomatoes [103], which may also be related to the dose used, the time of application, and the time of tissue collection.

Like the effect of AA on banana shelf quality [94], studies have shown that GB application also has a positive impact in the post-harvest life of commercial fruits. Immersion of hawthorn berries (*Crataegus monogyna*) in different GB concentrations and stored at 1 °C for 20 days suffered 25% less chilling injury compared to untreated controls. Application also caused significant increases in endogenous GB, AA, and proline levels, and raised activity levels of ROS scavenging enzymes (Razavi et al., 2018). Similar results were obtained in papaya (*Carica papaya*) immersed in GB after harvesting but prior to cold storage (6° for 40 days) [104].

Thus, GB not only alleviates damage produced by abiotic factors, but also enhances post-harvest life. Progress is being made in additional field studies that are identifying the species and genotypes that respond best to GB.

### 3.5. Alpha-Tocopherol (α-Toc)

Tocopherols are lipophilic compounds belonging to the vitamin E family. The four isomers α, β, γ, and δ play important roles in plant growth, stress responses, senescence, and signal transduction, among other processes [105]. Of these compounds, α-tocopherol (α-Toc) is the most abundant and is present in chloroplasts. One of its main functions is to prevent oxidative damage to thylakoid membranes by quenching and scavenging singlet oxygen (one α-Toc quenches approximately 120 singlet oxygen molecules) [106] and scavenging lipid peroxyl radicals, maintaining photosynthetic membrane integrity.

In recent years, the role of α-Toc in stress tolerance in crops has been examined. Most studies have investigated the beneficial effects of foliar α-Toc application during saline or drought stress. In a field experiment, Semida et al. reported a significant increase in growth parameters such as shoot length, number of leaves, leaf area, shoot DW, and FW in onion (considered a salt-sensitive crop) grown under saline condition [107] when α-Toc was used. The time of application also influences the effects of α-Toc on plant development. Lalarukh et al. [108] treated seeds of two sunflower (*Helianthus annuus*) varieties with different α-Toc concentrations and grew them in pots under NaCl treatment. Given that sunflower is moderately tolerant to salt stress, it is a good candidate for growing in saline soils. α-Toc seed priming increased shoot and root FW and shoot and root length, although the precise effect depended on both the cultivar and the metabolite concentration. It also improved yield: the authors reported up to 33 and 16% increases in total achene weight and hundred achene weights, respectively [109]. Similar results were obtained in wheat and mungbean, both grown under natural field conditions, when this metabolite was applied to plants under a water-stress regime [110,111]. Regarding yield improvement, α-Toc increased the number of seeds, pods, weight of ripened pods, and weight of 100 mungbean seeds [111]. In wheat, yield per plant increased by 18% when α-Toc was applied under water-stress conditions, mirrored by improvements in nutritional quality due to rises in α-, β-, γ-tocopherols, phenolics, and flavonoids [110]. Foliar application of this secondary metabolite also raised chlorophyll *a* and *b* levels and antioxidant enzyme activities in mungbean, wheat, and onions under saline stress conditions [107,110,111].

α-Toc has thus proven to enhance growth parameters and yield in crop species grown under field conditions and drought, indicating a potential use in commercial formulations. However, its beneficial effects in crops under other kinds of abiotic stress have yet to be addressed.

### 3.6. Melatonin

Melatonin (N-acetyl-5-methoxytryptamine) is a tryptophan derivative that was discovered in plants in 1995 [112,113]. Although its role in physiological processes in mammals has been extensively described [114], its function and action mechanism in crops is still being elucidated. Melatonin participates in different developmental processes, such as seed germination, growth regulation, fruit ripening, and rhizogenesis, among others [115]. Because melatonin also presents natural antioxidant capacity and delays leaf senescence, it has been proposed as a potential biostimulant for crops. An excellent review on melatonin and its function in the regulation of the plant antioxidant machinery in stressed conditions was recently published [116], thus, this section will focus on physiological parameters.

Melatonin treatment of salt-stressed tomato plants in growth chamber conditions improved growth parameters, generated higher chlorophyll *a* and *b* accumulation, and increased the activities of carbonic anhydrase and rubisco [117]. This is consistent with previous reports in other species, where melatonin treatment downregulated the expression of chlorophyllase and pheophorbide a oxygenase, two enzymes involved in chlorophyll degradation [118], thus improving photosynthetic efficiency of plants under cold stress [119].

Moreover, melatonin application increased proline levels and the activity of the enzyme required for its biosynthesis, P5CS [117]. Crosstalk of melatonin with other phytohormones, including auxin, cytokinin, gibberellins, abscisic acid, ethylene, jasmonic acid, and salicylic acid, has been determined [120]. It is a ROS scavenger and also increases the activities of antioxidant enzymes and metabolite contents [119,121,122]. Taking advantage of this crosstalk might optimise its use as a biostimulant for crops cultivated under abiotic stress. Melon seedlings (*Cucumis melo*) subjected to cold stress in growth chamber conditions presented higher chlorophyll *a* and *b* contents, net photosynthetic rate, stomatal conductance, and transpiration rate when treated with melatonin, with an optimum dose of 200 µM [123].

Other benefits attractive to the agronomical industry may be associated with abiotic stress tolerance. For instance, table grape bunches (*Vitis vinifera*) treated with melatonin presented a significantly lower berry abscission rate and a reduced rotten index, as well as more extended post-harvest life [124].

Whether melatonin should be considered a new phytohormone or a metabolite is currently under discussion [115]. Nevertheless, field trials evaluating the effects of melatonin on crops subjected to abiotic stress should be carried out in order to assess its potential impact on the yield of other species.

## 4. Conclusions and Future Prospects

Application of primary and secondary metabolites naturally found in plants has proven to be effective at ameliorating abiotic stress damage in crops. A variety of effects are observed in applying chemically diverse natural compounds. Some effects are common to most crop species studied, whereas others are species and even genotype specific (such as GB application in wheat [102] and Thin, 2015). Moreover, certain metabolites might perform better at alleviating specific types of stress: GSH seems to induce more tolerance to heavy metal stress, especially Hg [52]. Some of the differences observed within application of the same metabolite can be attributed to experimental design, the duration of stress, and the stage of plant development at the time of treatment. However, as Paracelsus noted, “the dose makes the poison”, and plant metabolites are not the exception (Paracelsus, “The Third Defense”). Careful consideration must be given to the dosage used, as excess of a certain metabolite might have detrimental effects on plant growth and yield.

An interesting development is the application of combinations of plant metabolites. For example, the use of ascorbic acid (AA) in addition to citric acid (CA) (2:1) had a particularly beneficial effect on stressed plants. In salt-stressed cowpea (*Vigna sinensis*; 75 mM) foliar application of AA + CA increased parameters FW and DW by 120 and 131%, respectively, as well as the number of pods and yield per plant, compared with the control (salt-stress alone). Improvements in photosynthetic pigment levels and the content of nitrogen/phosphorus/potassium were also observed, leading to greater tolerance to salt stress [125]. A similar effect occurred in apple (*Malus x domestica* cv. Red Spur); AA + CA treatment increased total anthocyanin content, the total antioxidant capacity, and the activity of antioxidant enzymes (CAT), and decreased activity of polyphenol oxidase (responsible for flesh oxidation), resulting in improved nutritional quality and fruit colour [126]. These studies show that the combination of two natural metabolites (in these cases, AA + CA) is beneficial for plant development and enhancing the commercial characteristics of crops.

Although the use of natural primary and secondary metabolites has proven useful to improve abiotic stress tolerance in crops, there is a limitation to their chemical diversity. To further broaden the spectrum of compounds that can be used for this purpose, chemical genomics can be used. As the discovery of new compounds to improve abiotic stress tolerance in crops is needed, this technique has been applied to the challenge of identifying novel molecules that promote plant growth under stress conditions. Chemical genomics allows the screening of thousands of compounds from chemical libraries, based on leaf and root growth parameters in a cost- and time-efficient manner [127] [128]. This approach has proven to be effective for evaluating and selecting new compounds that could modulate plant growth under stress conditions in crops [127], and the further exploration of chemical genomics will no doubt be invaluable for progress in this field.

For several of the metabolites presented, the scalability and cost of application in crops cultivated in the field must still be evaluated. Nevertheless, in general, they are readily available for widespread implementation, and a concentrated formula could be applied in the field with the application equipment that producers already use. Although field experiments are being carried out to test the effectiveness of plant metabolites on yield, there is still the need to evaluate their potential interactions with other chemicals (both natural and artificially added) in irrigation waters. Furthermore, the availability and production costs should be built into the equation to determine their viability as commercial products. Finally, there are salt-susceptible crop varieties that present unique or customer-valued attributes, and for these reasons, they cannot be replaced, or it is difficult to do so. In such cases, the application of different metabolites (alone or in combination with others) will allow its cultivation in saline soil conditions, maintaining its yield.
